# Can medicinal mushrooms have prophylactic or therapeutic effect against COVID‐19 and its pneumonic superinfection and complicating inflammation?

**DOI:** 10.1111/sji.12937

**Published:** 2020-07-29

**Authors:** Geir Hetland, Egil Johnson, Soosaipillai V. Bernardshaw, Bjørn Grinde

**Affiliations:** ^1^ Department of Immunology and Transfusion Medicine Oslo University Hospital (OUH) Oslo Norway; ^2^ Institute of Clinical Medicine University of Oslo Oslo Norway; ^3^ Department of Gastrointestinal and Pediatric Surgery Oslo University Hospital Oslo Norway; ^4^ Bergen Accident and Minor injury Department Haukeland University Hospital Bergen Norway; ^5^ Norwegian Institute of Public Health Oslo Norway

**Keywords:** COVID‐19 infection, inflammation, medicinal mushrooms, pneumococcal infection

## Abstract

Medicinal mushrooms have documented effects against different diseases, including infections and inflammatory disorders. The related *Basidiomycota Agaricus blaz*ei Murill (AbM), *Hericium erinaceus* (HE), and *Grifola frondosa* (GF) have been shown to exert antimicrobial activity against viral agents, Gram‐positive and Gram‐negative bacteria, and parasites in vitro and in vivo. Since the mechanism is immunomodulatory and not antibiotical, the mushrooms should be active against multi‐drug resistant microbes as well. Moreover, since these *Basidiomycota* also have anti‐inflammatory properties, they may be suited for treatment of the severe lung inflammation that often follows COVID‐19 infection. An AbM‐based mushroom extract (Andosan™), also containing HE and GF, has been shown to significantly reduce bacteraemia and increase survival in mice with pneumococcal sepsis, and to improve symptoms and quality of life in IBD patients via an anti‐inflammatory effect. Hence, such mushroom extracts could have prophylactic or therapeutic effect against the pneumonic superinfection and severe lung inflammation that often complicates COVID‐19 infection. Here, we review antimicrobial and anti‐inflammatory properties of AbM, HE and GF mushrooms, which could be used for the battle against COVID‐19.

## 1 INTRODUCTION

Edible mushrooms, especially of the *Basidiomycetes* family, have had a long and apparently successful medicinal use based on empiric observations, foremost in traditional Chinese and Japanese medicine. *Basidiomycetes* mushrooms such as *Agaricus blazei* Murill (AbM), *Ganoderma lucidum, Hericium erinaceus* (HE) and *Grifola frondosa* (GF) are consumed as immune response modifiers for prevention of cancer, or as nutritional support during chemotherapy, and for chronic inflammatory conditions such as hepatitis and other diseases.^1^ Substances derived from fungi include antibiotics, for example penicillin and griseofulvin, and the immunosuppressant, cyclosporine A, which is crucial in organ transplantation. Substances have been detected in higher *Basidiomycetes* mushrooms that have a range of therapeutic effects,^2^ including chemically highly diversified anti‐inflammatory compounds, such as polysaccharides,^3^ terpenoids,^4^ phenolic compounds,^5^ glycerides^6^ and other low molecular weight molecules.^7^

Effects of AbM on infection, inflammation and tumour have been reviewed previously in SJI, including that of the AbM‐based mycelium extract, Andosan™, which also contains HE (15%) and GF (3%).^8^ It has been used in three placebo‐controlled randomized clinical trials as supplement to regular treatment for inflammatory bowel disease (IBD); ulcerative colitis (UC) (50 patients) and Crohn's disease (CD) (50 patients),^9^, ^10^, ^11^ multiple myeloma (MM)^12^ (40 patients) and pollen allergy and asthma^13^ (60 blood donors) without adverse effects. It reduced proinflammatory cytokines and improved symptoms and quality of life in IBD patients,^9^, ^10^, ^11^ reduced allergy and asthma symptoms, specific IgE and basophil sensitivity in allergics,^13^ and increased IL‐1 receptor antagonist (IL‐1ra), IL‐7, T regulatory cells, dendritic cells (DCs) and expression of Ig, Killer Ig receptors (KIRs) and HLA genes in MM patients.^12^ Moreover, since the beginning of this millennium there have been quite a few other reports on antimicrobial (Table 1, 2) and anti‐inflammatory (Table 3) effects of medicinal mushrooms such as AbM, HE and GF.

**Table 1:** Antiviral effects of AbM, HE and GF (
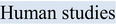
)

Viral agent	Experimental model	Mushroom product	Antiviral effect/Mechanism	Author	References
WEE virus	In vitro, cell cultures	AbM extract	Anti‐cytopathic effect induced by WEE virus in Vero cells	Sorimachi et al, 2001	[25]
Polio virus	In vitro	AbM extract	Reduced virus replication	Faccin et al, 2007	[26]
HBV	HEp G2 cells	GF extract	Induction of endogenous antioxidant enzyme	Gu et al, 2006	[29]
	Patients with chron. Infection (n = 4)	AbM extract	Normalized liver function	Hsu et al, 2008	[27]
HCV	Patients with chron. Infection (n = 5)	Mycelium extract incl. HE, GF, (Andosan) p.o.	Slight reduction (not significant)	Grinde et al, 2006	[28]
HSV‐1 or HSV‐2	In vitro, and in Mice	GF protein, topical admin.	Reduced virus production	Gu et al, 2007	[30]
	HEp‐2 cultures	AbM polysaccharide and beta‐glucan	Inhibition	Minari et al, 2011	[31]
	HEp‐2 cultures	AbM polysaccharide	Inhibition	Yamamoto et al, 2013	[32]
	Mice, ocular, cutaneous and vaginal infections	AbM mycelial polysacc., p.o.	Reduced topical infections	Cardozo et al, 2013	[33]
Influenza virus	In vitro	AbM metabolites	Direct antiviral action	Avtonomova et al, 2014	[35]
	Plaque formation inhib. test	AbM extract	Inhibition	Eguchi et al, 2017	[36]
Enterovirus 71	In vitro	GF polysaccharide, p.o.	Blocked viral replication	Zhao et al, 2016	[34]
Muscovy duck reovirus	Ducklings	HE polysaccharide	Restor. of injured mucosal immunity	Wu et al, 2018	[37]
Dengue virus	In vitro	HE	Inhibition	Ellan et al, 2019	[38]

**Table 2:** Antimicrobial effects of AbM, HE and GF against bacteria and parasites

Microbe	Experimental model	Mushroom product	Antimicrobial effect/Mechanism	Author	References
*Streptococcus pneumoniae* serotype 6	Mice, sepsis	Mycelium extract incl. HE, GF (Andosan) p.o.	Reduced bacteraemia, Increased survival	Bernardshaw et al, 2005	[39]
*Streptococcus mutans*	In vitro	Erinacine from HE	Suppressed mutacin synthesis	Premnath et al, 2018	[43]
Faecal Gram neg. bacteria	Mice, sepsis	Mycelium extract incl. HE, GF, (Andosan), p.o.	Reduced bacteraemia, Increased survival	Bernardshaw et al, 2006	[40]
*Pseudomonas aeruginosa*	In vitro	AbM	Antiquorum sensing	Sokovic et al, 2014	[44]
*Pseudomonas* sp, pathogen opportunists	Plaque formation inhib. test	GF furanone	Inhibition	He et al, 2016	[48]
*Helicobacter pylori*	In vitro	HE extract	Inhibition	Liu et al, 2016	[49]
	In vitro & Mouse colonization assay	HE extract	Inhibition	Wang et al, 2019	[51]
Microbiota	Mice	HE	Improved colonic health	Wang et al, 2018	[52]
*Leishmania*	Mice, visceral L	AbM extract	Th1 response	Valadares et al, 2012	[45]
	Mice	AbM extract	Th1 response	de Jesus Pereira et al, 2015	[46]
*Plasmodium berghei*	Mice, cerebral malaria	AbM	Improved outcome	Val et al, 2015	[47]

**Table 3:** Anti‐inflammatory effects of AbM, HE and GF (
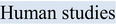
)

Product, Applic.	Study in, of	Effects	Mechanism	Author, Year	References
Mycelium extract incl. HE, GF, (Andosan) p.o.	Healthy Volunteers (n = 10)	Predominantly anti‐inflammatory effect	↓Proinflammatory cytokines	Johnson et al, 2009	[21]
Mycelium extract incl. HE, GF, (Andosan) p.o.	Healthy Volunteers (n = 8)	Antioxidant effect	↓iROS prod. & adhesion molec. expressio in MΦ & granulocytes	Johnson et al, 2012	[24]
Mycelium extract incl. HE, GF, (Andosan) p.o.	IBD patients (50 UC & 50 CD)	Improved symptoms & QoL, espec. in UC	↓ Proinflammatory effect	Therkelsen et al, 2016a‐c	[9, 10, 11]
AbM extract, p.o.	Rats, Pulmonary inflammation	↓ Lung damage induced by carcinogen	Attenuation of pulmonary inflammation & gross consolidation	Croccia et al, 2013	[52]
HE mycelium & erinacine A, p.o.	Rats, brain ischaemia	Protection against brain ischaemia injury induced neuronal cell death	Inhibition of iNOS/P3 MAPK Reduced IL‐1β, IL‐6, TNFα, nerve growth properties	Lee et al, 2014	[53]
AbM extract fractions	Mice, cerebral malaria	Improved consequence of cerebral malaria	↓ TNFα, IL‐6, IL‐1β Antimalarial activity	Val et al, 2015	[47]
HE extract & polysacc., p.o.	Rats, IBD	Improved damages in colonic mucosa of induced IBD	↓MPO activ., NF_K_B, TNFα, ↑T cell activ. Growth of beneficial gut bacteria and improved host immunity	Diling et al, 2017	[54]
HE polysaccharide, p.o.	Mice, Colitis	Attenuation of colitis, reversing of gut dysbiosis	Downregulation of oxidative stress & inflamm.‐related signalling pathways, Maintaining intestinal barrier	Ren et al, 2018	[55]
AbM dry feed, p.o.	Mice, non‐alcoholic steato‐hepatitis	Prevention	Prevention of oxidative stress	Nakamura et al, 2019	[56]
Erinacine A‐enriched HE mycelia, p.o.	Aged Mice	Increased longevity	Induction of endogenous antioxidant enzymes	Li et al, 2019	[58]

The outbreak of a novel coronavirus (SARS‐CoV‐2) (COVID‐19)–induced disease in China that causes serious respiratory illness, was declared a pandemic by the World Health Organization on 11 March 2020. Most of those who got sick from the infection, developed lymphopenia and pneumonia and in severe cases also high levels of proinflammatory cytokines.^14^ This is similar to the 'cytokine storm' observed in SARS,^15^, ^16^ which gives rise to viraemia and inflammatory lung injury and may be followed by multi‐organ failure and death. In some cases, this pathogenesis will be enhanced by secondary bacterial superinfection in the lung as well as development of septicaemia. Immune dysregulation may play an important role in many viral diseases such as respiratory syncytial virus infection, where a disease‐enhancing inflammation similar to the COVID‐19 situation is dependent on the immune response in the airways mucosa.^17^ In viral infections, Treg and Th17 cells have a complex interaction in which Tregs may inhibit immune activation and subsequent disease progression and maintain immune homeostasis, while Th17 cells will induce immune activation and propagate the inflammation.^18^

As the COVID‐19 pathogenesis is unknown, similar to that of SARS, there are no approved drugs for the disease and vaccines are yet to be developed. Corticosteroids that otherwise are used for treatment of acute respiratory distress syndrome and severe lung injury,^16^ strongly inhibit antiviral immunity and may be counter‐indicated. An inhibition of a proximal immune response event such as activation of IFN‐related pattern recognition receptors (PRRs) that are activated by pathogen‐ (PAMPs) and danger‐associated molecular patterns (DAMPs) at the mucosa,^19^ would seem unwise because of its general host defence regulatory function. Therefore, targets should be limited to proinflammatory and Th2 cytokines, such as oxygen radicals, TNFα, IL‐1, IL‐4, IL‐6, IL‐8 and IL‐21 production.^20^ These, except for the IL‐21 that has not been examined, are the very same effector arms that are counteracted by Andosan™ treatment.^21^, ^22^, ^23^, ^24^

The aim of this article was to review possible effects that the much used and related medicinal mushrooms AbM, HE and GF might have against COVID‐19 infection and its complications. Selection criteria were inclusion of PubMed/Medline indexed articles on antimicrobial and anti‐inflammatory effects with these mushrooms.

## 2 ANTIVIRAL EFFECTS OF ABM, HE AND GF

AbM has been shown to counteract the cytopathic effect induced by Western Equine Encephalitis (WEE) virus on VERO cells in vitro,^25^ and in a plaque reduction assay with poliovirus to reduce the number of plaques suggestively by acting in the initial stage of viral replication (Table 1).^26^ In patients with chronic hepatitis B virus (HBV) and C virus (HCV) infection, AbM extracts have been found to normalize liver function,^27^ and to slightly decrease HCV plasma load.^28^ Also antiviral effect of GF alone or combined with IFNα has been demonstrated against HBV in HepG2 cells, in which HBV DNA was inhibited.^29^

There are several reports regarding mushroom treatment of herpes virus 1 (HSV‐1) and 2 (HVS‐2): a protein isolated from GF inhibited HSV‐1 replication in vitro and reduced severity of the viral infection upon topical administration in a mouse model.^30^ Further, AbM polysaccharides inhibited HSV‐1 infection in HEp‐2 cell cultures.^31^, ^32^ Another AbM mycelium polysaccharide given orally to mice, reduced ocular, cutaneous and vaginal (HSV‐2) infections by inhibition of virus attachment, entry and cell‐to‐cell spreading as shown by plaque reduction assay.^33^ Suggestively, this occurred through interference with early events of viral penetration.^31^ Yet, another GF polysaccharide was shown to block replication of enterovirus 71 (EV‐71) ‐ the major agent for foot, hand and mouth disease ‐ suppress viral protein expression and exhibits apoptotic activity in vitro.^34^

With respect to influenza, one report found that AbM metabolites had direct antiviral effect against influenza virus among others in vitro,^35^ and another reported inhibition by AbM extract against H1N1 influenza virus in a plaque formation test after the viral invasion of host cells.^36^ Also, antiviral effects have been proved for HE: it was effective against intestinal damage of Muscovy duck reovirus in ducklings, in which injured mucosal immunity was restored.^37^ Moreover, HE is reported to counteract Dengue virus infection in vitro as shown by inhibition of attachment and penetration in plaque reduction assays and reduction in viral gene expression.^38^

## 3 ANTIMICROBIAL EFFECTS OF ABM, HE AND GF AGAINST BACTERIA AND PARASITES

**Figure 1:**
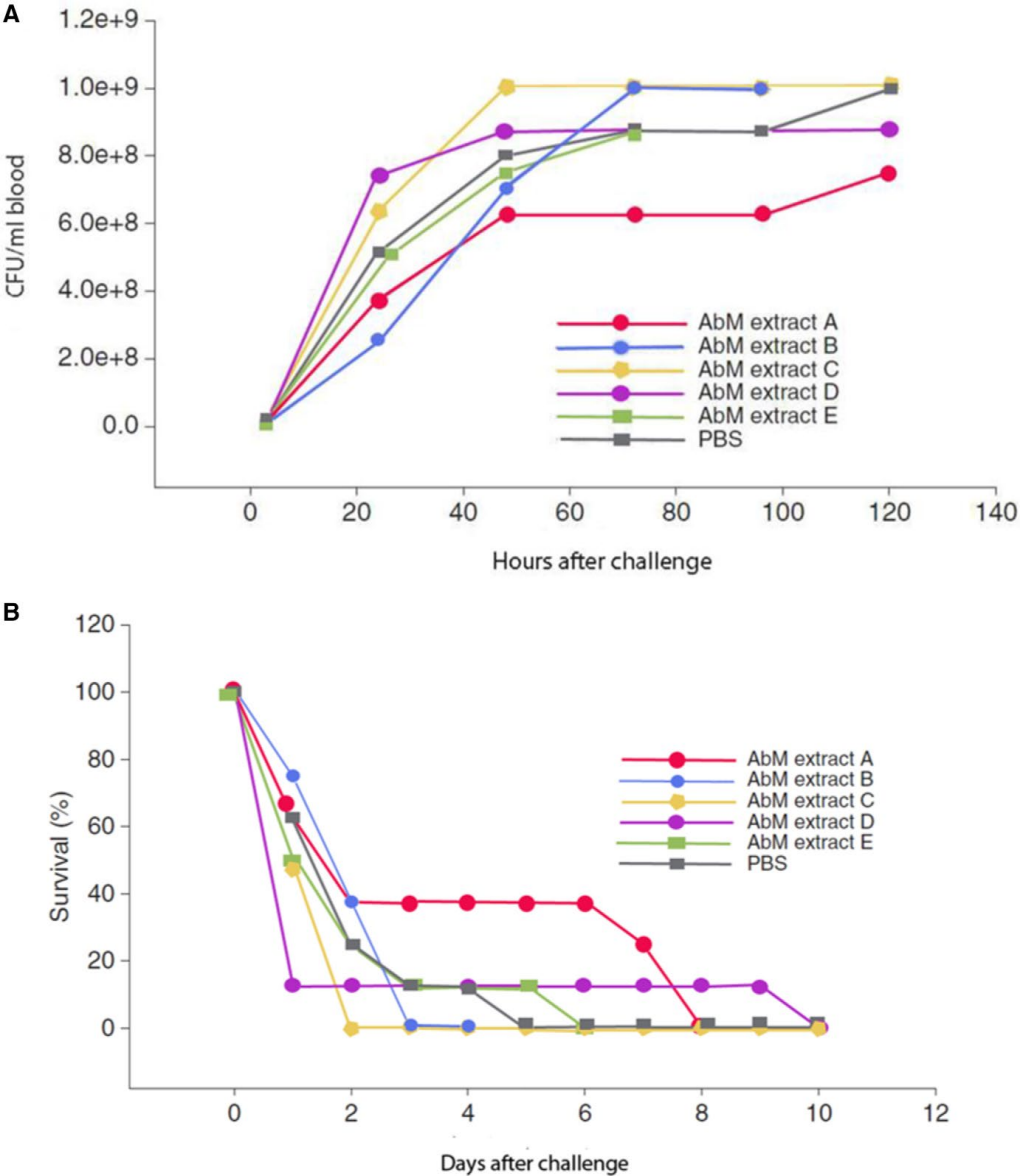
Bacteraemia (A) (no. of CFU after plating of 25ul blood drawn daily from the animals’ lateral femoral vein) and survival (B) of mice (8 per group) challenged i.p with *Streptococcus pneumoniae* serotype 6B 2h after oral administration (gavage) of different AbM extracts. Extract A is Andosan™, which was the only extract that showed significant differences (*P* < .05) compared with saline control. Control was phosphate‐buffered saline (PBS) (Modified from Ref. [4] with permission from Scand J Immunol for republication)

There are several publications regarding antibacterial and antiparasitic properties of AbM (Table 2). We reported 15 years ago that the AbM‐based extract, Andosan™, when given orally 1 day before or at time of intraperitoneal (i.p.) inoculation of bacteria, significantly reduced bacteraemia and increased the animals' survival in two lethal bacterial sepsis models in mice.^39^, ^40^ One model was with Gram‐positive pneumococci (*Streptococcus pneumonia* serotype 6B)^39^ that tend to give pneumonia as superinfection in elderly COVID‐19 infected patients.^41^ The other sepsis model was with a suspension of air‐exposed mouse fecalia, predominantly containing Gram‐negative bacteria^40^ and their toxins, which are feared culprits for sepsis development with its life‐threatening complications from organ failure.^42^ In the pneumococcal infection model, also effects of other Japanese AbM extracts were studied in a blinded fashion after administration orally 2h before i.p. bacterial challenge. However, only Andosan™ gave a statistically significant (*P* < .05) decrease in bacteraemia (>1 log, day 10) and increase in survival rate; 38% (3/8) survival day 6 in Andosan™ group and none day 5 in saline controls (Figure 1).^8^ In fact, whereas 50% or 40% of the mice survived when given Andosan™ by gavage 24 hours before or at time of i.p. injection of the bacteria, respectively, only 13% of the saline gavage controls survived the 10 days of experimentation in follow‐up experiments.^39^ In the more lethal faecal sepsis model, 33% of mice given Andosan™ 24 hours prior to i.p. bacterial challenge survived during the 7 days experiment in contrast to the saline controls that were all dead on day 3.

^40^

When testing natural products against synthesis of the bacteriocin mutacin by *Streptococcus mutans* in dental plaque biofilm, it was found to be inhibited by erinacine C from HE.^43^ Quorum sensing plays an important role for virulence, biofilm formation and survival of many pathogenic bacteria, including Gram‐negative *Pseudomonas aeruginosa*.^44^ Interestingly, an AbM extract has been shown to have antiquorum sensing effect as demonstrated by reduction of virulence factors of *P aeruginosa* and its biofilm forming capability, which may be used as weapon against such pathogens.^44^ With regard to parasitic infections, AbM has been shown to counteract murine visceral leishmaniasis^45^ by induction of a Th1 immune response,^46^ and also to improve the consequence of cerebral malaria, by reduction of *Plasmodium berghei* fluorescence labelled red blood cells in blood of mice, following their i.p. instillation.^47^

A GF furanone is found to inhibit opportunistic *Pseudomonas* sp. pathogens in a plaque formation test.^48^ There are two reports on inhibitory effect of HE extract against *Helicobacter pylori* in vitro.^49^, ^50^ HE also affected microbiota, which resulted in improved colonic health.^44^

## 4 ANTI‐INFLAMMATORY EFFECTS OF ABM, HE AND GF

Johnson et al reported that Andosan™ had predominantly anti‐inflammatory effect in vivo, as demonstrated by systemic reduction in proinflammatory cytokines^21^ and antioxidant effect in peripheral leucocytes (Table 3).^24^ Another AbM extract given orally to rats with carcinogen‐induced lung damage, attenuated the pulmonary inflammation and ensuing gross pulmonary consolidation.^52^ As mentioned, AbM did improve cerebral inflammation from malaria.^47^ Moreover, HE mycelium and HE‐derived erinacine A protected against neural cell death induced by brain ischaemia in a rat model by inhibiting iNOS and MAP kinase, proinflammatory cytokines TNFα, IL‐1β and IL‐6, and promoting nerve growth properties.^53^

In a randomized clinical study (RCT), multiple myeloma patients undergoing high‐dose chemotherapy and given add‐on placebo‐controlled treatment with the AbM‐based Andosan™ for 2 months, were found to have the following immunomodulatory effects^12^: Reduced IL‐1ra levels in plasma and increased T regulatory cells indicating anti‐inflammatory effect, and increased plasmacytoid DC in blood and increased expression of genes in bone marrow aspirate at end of study vs before for KIRs and MHC antigens, the latter being important for antigen presentation. In another placebo‐controlled RCT with IBD patients,^9^, ^10^ Therkelsen et al showed that Andosan™ given orally for 3 weeks reduced symptoms and increased quality of life especially of the patients with ulcerative colitis,^9^ by an anti‐inflammatory mechanism.^13^ In a rat IBD model, also HE extract and isolated polysaccharide were shown to improve IBD‐induced colonic mucosa damage by reducing MPO activity.^54^ In colonic mucosa, also NF_Κ_B and TNFα expression was decreased and T cells were activated and growth of beneficial gut bacteria was promoted.^54^ Additionally, a HE polysaccharide was shown to attenuate colitis in mice by reversing gut dysbiosis from potentially proinflammatory microbes, for example *Corynebacterium* and *Staphylococcus*, to potentially anti‐inflammatory microbes, for example *Bacteroides* and *Bifidobacterium*.^55^ The mechanism was downregulation of oxidative stress and inflammatory signalling pathways and maintenance of the intestinal barrier by blocking phosphorylation of NF_Κ_B, and protein kinases MAPK and Act in mice with induced colitis.^55^

Moreover, AbM dry feed has been shown to prevent non‐alcoholic steato‐hepatitis (NASH) in a mouse model by preventing oxidative stress.^56^ Similarly, a GF polysaccharide was shown to ameliorate lipid metabolic disorders in rats by beneficial regulation of microbiota.^57^ Interestingly and in line with this, it was found that erinacine A‐enriched HE mycelia given orally to aged mice, increased their longevity by induction of endogenous antioxidant enzymes.^58^

## 5 MECHANISM OF ACTION—IMMUNOMODULATION AND DEFENCE AT THE MUCOSA

Immunomodulating β‐glucans constitute the main part of the cell wall in fungi, including AbM, HE and GF mushrooms.^59^ Such polysaccharides have been found to have anticancer and anti‐infection effects when given i.p. in mouse models.^59^, ^60^, ^61^ Previously, we have also shown that yeast β‐glucan given orally can protect against systemic *S. pneumoniae* infection in mice.^62^ Moreover, owing to AbM‐induced humoral and cellular responses, they are capable of adjuvating positive effects of hepatitis B virus and foot‐and‐mouth disease DNA vaccines in mice.^63^, ^64^

AbM, HE and GF share PAMPs and DAMPs with other highly poisonous and health‐threatening fungi and macrofungi. Accordingly, this must be the reason for the observed strong and rapid engagement of innate immunity and subsequent skewing of adaptive immunity from Th2 towards Th1 responses in the host when encountering edible and harmless mushrooms such as AbM, HE and GF.^22^, ^45^, ^47^, ^65^ PRR of the innate immune system such as TLR2, dectin‐1 and CR3,^66^, ^67^, ^68^ recognize immediately PAMP such as β‐glucans from the main cell wall in mushrooms and fungi. TLRs and nucleotide‐binding oligomerization domain (NOD)‐like receptors are the two main PRR, which interact, for example in *Aspergillus fumigatus* infection.^69^ Furthermore, β‐glucan activation of dendritic cells to IL‐1β production occurs subsequent to NOD inflammasome activation.^70^

*Candida albicans* exists as a benign, commensal member of microbiota on mucosal surfaces in most humans, where it triggers numerous innate responses, but overgrowth can give localized mucosal or systemic infection.^19^ Interestingly, *C. albicans* hyphae of the mycelium evoke PRR activation by their DAMPs and stimulate production of antimicrobial peptides^19^ by epithelial and innate immune cells,^71^, ^72^ whereof defensins are the largest group.^73^ Other properties than just their β‐glucans probably are important triggers of defensins and other antimicrobial peptides. In fact, it was a surprise when it recently was revealed that the β‐glucan content of Andosan™ was very low, probably due to its source being mycelia and not fruiting body.^74^ Nevertheless, this mixed mushroom extract did stimulate TLR2 in monocytic cells.^75^ Such AbM stimulation is shown to promote differentiation of M2 to M1 cells and induce a potent antitumour effect.^76^ AbM also stimulates the expression of NKG2D/NCR cell surface receptors on NK cells.^77^

Since port of entry for most non‐vector‐borne viruses is the mucosa, which also is the body surface exposed to the mushroom extracts upon intake, in vitro virus studies referred to (see Table 1) are quite similar to the in vivo situation. Hence, AbM and GF may very well have similar antiviral effect in vivo against polio virus and EV‐71, respectively, as demonstrated in vitro.^26^, ^34^ Enteroviruses such as polio and EV‐71 infect by the faecal‐oral route and target gastrointestinal epithelium where they are detected and trigger innate immunity signalling, which they yet are masters in evading.^78^ This deregulation of inflammatory responses that results in a cytokine storm may play a critical role in pathogenesis of EV‐71 pulmonary oedema^79^ and that of COVID‐19 infection as well.^15^, ^16^, ^17^ Hence, one may assume that AbM and GF also could counteract the COVID‐19 inflammatory lung injury.

Regarding HSV‐1 and HSV‐2 infections the host fails in initiating an effective early innate antiviral response and DC function, which should be targets for prophylactic strategies for preventing infections with these viruses.^80^ This may be the very same mechanism(s) as for the reported antiherpetic action of a GF protein and a AbM mycelial polysaccharide in vivo.^30^, ^33^ Moreover, the amelioration by AbM of the WEE virus‐induced cytopathic effect demonstrated *in vitro* ^25^ may reflect the antiviral effect against flaviviruses such as dengue virus also shown for HE.^39^ In Muscovy duck reovirus infection, there was a net loss of beneficial bacteria that produce short chain fatty acids (SCFA) and compensatory proliferation of pathogenic bacteria (Gram‐negative *Enterobacteriaceae*). This disruption of intestinal microbiota then results in severe pathology of intestinal mucosa and acute diarrhoea.^81^ The injured mucosa and its immunity could be restored by HE, which was effective against this viral disease in ducklings.^37^ Furthermore, oral intake in mice of Andosan™ is shown to promote growth of *Bacteroides*, potentially anti‐inflammatory microbes,^57^ and production of SCFA (commun. prof. T. Ogita, Shinshu Univ, Nagano, Japan). This is also supported by our previous finding in the Gram‐negative faecal sepsis mouse model of significantly increased survival after oral treatment with Andosan™.^39^ Moreover, the antiviral effect of AbM on influenza virus^35^, ^36^ is interesting because influenza corona viruses can give similar lung problems as COVID‐19.

Although there was only insignificant reduction of HCV load in a few patients with chronic HCV infection who ingested Andosan™ for a week, there was an increased expression in peripheral mononuclear leucocytes of genes related to G protein‐R signalling, cell cycling and transcriptional regulation.^28^ G protein–coupled receptors for chemotaxins such as IL‐8 chemokine, leukotriene 4B, the complement activation product C5a and bacteria‐derived formyl peptides are associated with inflammation and microbial defence.^82^

Since complement is involved in the pathogenesis of several of the diseases, due to self‐attack or contribution to the overall pathology,^83^, ^84^, ^85^ which the mushrooms contained in Andosan™ have proposed or documented health effects against, the mechanism of action of Andosan™ may very well be linked to complement activity. Examples of diseases with beneficial effect of these mushrooms are as follows: Alzheimer's disease,^86^ IBD,^9^, ^10^ bacterial infections,^39^, ^40^ malaria,^47^ and allergy and asthma.^13^ This is supported by AbM’s ability to activate the alternative pathway of complement.^87^ Also, there is new evidence for intracellular complement—the complosome—that is thought to be involved in immune cell regulation and metabolism in T cells and monocytes.^88^ This may further explain the involvement of complement in pathogenesis of such quite different diseases. The existence of intracellular complement was detected more than 30 years ago by the first and second authors of this paper who showed that mononuclear phagocytes could produce all components for a functional complement system.^89^, ^90^

## 6 ABM, HE, GF AND COVID‐19 INFECTION

In the current situation with a seemingly non‐curable pandemic at hand and where candidate drugs and vaccines just are in the testing stage, one must look at alternative prophylactic and therapeutic principles. One candidate is immune prophylaxis and/ or therapy by use of immunomodulatory mushrooms. The *Agaricomycota* among the *Bacidiomycetes* mushrooms, AbM, HE and GF, are well‐known medicinal mushrooms that have been used worldwide for a range of diseases in traditional medicine. In fact, many of those applications have been confirmed in preclinical and clinical studies. Focus has especially been on antitumour effects where cytotoxicity and apoptotic mechanism have been revealed. However, in addition to an anti‐inflammatory property, the mushrooms have also been found to induce enhanced Th1 cellular immune response, as demonstrated by increase in IFNγ, IL‐2 and IL‐12 cytokines.^22^, ^65^, ^91^

Cells participating in the Th1 response are activated NK cells and cytotoxic Th1 cells and γ/δ T cells, which besides tumour attack, also destroy virus‐infected cells. Moreover, γ/δ T cells play an important role in bridging the gap between innate and adaptive immunity, for example by being able to present antigen to conventional T cells, and they are predominantly localized in mucosa and epithelial sites,^92^ which are entry points for viruses. Type III interferons (IFN‐λ) are thought to be especially important in antiviral immunity.^93^, ^94^ Hence, the induction of the Th1 response by medicinal mushrooms could be tested as a novel modality for prophylactic and/or therapeutic measures against COVID‐19 infection, as well as against its hazardous bacterial superinfection. In fact, bacterial infection, and especially with *S. pneumonia,* is found in 43% of the admitted elderly COVID patients and in 82% of the dead.^41^ Besides elderly patients, also those with complicating underlying diseases such as chronic obstructive lung disease, cardiac diseases, diabetes and other chronic diseases with systemic affection^95^ are at risk.

In pneumococcal sepsis in mice caused by the strain *S. pneumonia* serotype 6B, initially isolated from a patient, the disease could be counteracted by Andosan™ both when given orally either before or simultaneously with i.p. challenge with the pneumococci.^39^ Hence, the extract seems to have both a prophylactic and a therapeutic effect against pneumococcal disease. Moreover, since the effect of Andosan™ was not antibiotical but immunomodulatory, this mushroom extract should be as effective against antibiotic‐resistant bacteria as against antibiotic‐sensitive bacteria. This aspect is especially interesting in context with the grave COVID‐19 situation that has been experienced in Northern Italy and Spain, where pneumonia superinfection with multi‐resistant bacteria may be an additive cause of death. Also, a more effective riddance of a bacterial superinfection would dampen the immune response and the ensuing inflammation that otherwise may complicate the COVID‐19 disease. Non‐digestible carbohydrates with prebiotic effect, such as β‐glucan polysaccharides from medicinal mushrooms, stimulate growth of gut microbes that are favourable to the host's health, and spur on the production of SCFA, which energize anaerobic gut microbes, suppress pathogens (eg *Salmonella* sp.) and improve host immunity.^95^, ^96^ In this context, the increased production observed of SCFA by microbiota would probably stabilize colonocytes by being their main nutritional substrate, especially β‐OH‐butyrate (70%), generally as well as in IBD. Such a trophical effect per se would normalize and equalize the physiological reaction to the body as such, which would benefit both healthy individuals prophylactically and patients therapeutically in the struggle against pathological agents, for example viral attacks such as from COVID‐19.

Besides in the bacterial sepsis models,^39^, ^40^ the mushroom mixed product Andosan™ has also given positive results both in murine models for allergy^22^ and colorectal cancer,^91^ where an increased T helper cell (Th) 1 immune response was found in both models in addition to a proinflammatory response (increased IL‐1β, MCP‐1, TNFα) in the latter. The proinflammatory response in mice is probably due to uptake of β‐glucans from the murine gut as opposed to the anti‐inflammatory effect in humans where β‐glucan is hardly taken up or to a lower degree,^97^ but may stimulate Peyer’s patches in the gut‐associated lymphoid tissue (GALT).^98^ Therefore, other absorbable less defined low molecular weight substances (eg flavonoids) with anti‐inflammatory and/or antioxidant activity probably contribute to this effect. In addition, in a placebo‐controlled RCT in individuals with pollen allergy and asthma, Andosan™ supplementation before the pollen season resulted in decreased symptoms, medication, specific plasma IgE levels and basophil sensitivity, owing to a skewing of Th1/Th2 cells towards the Th1 phenotype.^13^ The Th1 response is besides its induction of antitumoral and anti‐allergic activity, also driving an antiviral immune response as discussed.

In conclusion, from the literature it seems possible that the related medicinal *Basidiomycetes* mushrooms, AbM, HE and GF would have merit as prophylactic or therapeutic add‐on remedies in COVID‐19 infection, especially as countermeasures against a pneumococcal superinfection, even when caused by multi‐resistant bacteria, as well as for the immune overreaction and damaging inflammation that occurs with COVID‐19 attack.

## CONFLICT OF INTERESTS

Geir Hetland is a cofounder and shareholder of Immunopharma, Oslo, Norway. The other authors declare no commercial or financial conflict of interest. Immunopharma had no other role than providing Andosan™ free of charge for the studies referred to.

## AUTHOR CONTRIBUTIONS

Geir Hetland wrote the manuscript, and Egil Johnson, Soosaipillai Bernardshaw and Bjørn Grinde revised the manuscript. All authors have previously been much involved in several of the articles that this review is built upon.

## References

[1] Zhang JJ , Li Y , Zhou T , et al. Bioactivities and health benefits of mushrooms mainly from China. Molecules. 2016;21(7):938. 10.3390/molecules21070938 PMC6274515 27447602

[2] Wasser SP , Weis AL . Therapeutic effects of substances occurring in higher Basidiomycetes mushrooms: a modern perspective. Crit Rev Immunol. 2014;19:65‐96. 9987601

[3] Song HH , Chae HS , Oh SR , Lee HK , Chin YW . Anti‐inflammatory and anti‐allergic effect of Agaricus blazei extract in bone marrow‐derived mast cells. Am J Chin Med. 2012;40(5):1073‐1084. 10.1142/S0192415X1250079 22928836

[4] Dudhgaonkar S , Thyagarajan A , Sliva D . Suppression of the inflammatory response by triterpenes isolated from the mushroom Ganoderma lucidum. Int Immunopharmacol. 2009;9(11):1272‐1280. 10.1016/j.intimp.2009.07.011 19651243

[5] Moro C , Palacios I , Lozano M , et al. Anti‐inflammatory activity of methanolic extracts from edible mushrooms in LPS activated RAW 264.7 macrophages. Food Chem. 2012;130(2):350‐355. 10.1016/j.foodchem.2011.07.049

[6] Han C , Cui B . Pharmacological and pharmacokinetic studies with agaricoglycerides, extracted from Grifola frondosa, in animal models of pain and inflammation. Inflammation. 2012;35(4):1269‐1275. 10.1007/s10753-012-9438-5 22327864

[7] Wang J , Liu YM , Cao W , Yao KW , Liu ZQ , Guo JY . Anti‐inflammation and antioxidant effect of Cordymin, a peptide purified from the medicinal mushroom Cordyceps sinensis, in middle cerebral artery occlusion‐induced focal cerebral ischemia in rats. Metab Brain Dis. 2012;27(2):159‐165. 10.1007/s11011-012-9282-1 22327557

[8] Hetland G , Johnson E , Lyberg T , Bernardshaw S , Tryggestad AMA , Grinde B . Effects of the Medicinal Mushroom *Agaricus blazei* Murill on Immunity, Infection and Cancer. Scand J Immunol. 2008;68:363‐370. 10.1111/j.1365-3083.2008.02156.x 18782264

[9] Therkelsen SP , Hetland G , Lyberg T , Lygren I , Johnson E . Effect of a Medicinal Agaricus blazei Murill‐Based Mushroom Extract, AndoSan™, on Symptoms, Fatigue and Quality of Life in Patients with Ulcerative Colitis in a Randomized Single‐Blinded Placebo Controlled Study. PLoS One. 2016;11(3):e0150191. 10.1371/journal.pone.0150191 26933886 PMC4774976

[10] Therkelsen SP , Hetland G , Lyberg T , Lygren I , Johnson E . Effect of the Medicinal Agaricus blazei Murill‐Based Mushroom Extract, AndoSanTM, on Symptoms, Fatigue and Quality of Life in Patients with Crohn's Disease in a Randomized Single‐Blinded Placebo Controlled Study. PLoS One. 2016;11(7):e0159288. 10.1371/journal.pone.0159288 27415795 PMC4944955

[11] Therkelsen SP , Hetland G , Lyberg T , Lygren I , Johnson E . Cytokine levels after consumption of a medicinal Agaricus blazei murill‐based mushroom extract, AndoSan™, in patients with Crohn's disease and ulcerative colitis in a randomized single‐blinded placebo‐controlled study. Scand J Immunol. 2016;84(6):323‐331. 10.1111/sji.12476 27588816

[12] Tangen JM , Tierens A , Caers J , et al. Immunomodulatory effects of the Agaricus blazei Murill‐based mushroom extract AndosanTM in patients with multiple myeloma undergoing high dose chemotherapy and autologous stem cell transplantation. A randomized, double blinded clinical study. BioMed Res Int. 2015;2015:718539. 10.1155/2015/718539 25664323 PMC4312620

[13] Mahmood F , Hetland G , Nentwich I , Mirlashari MR , Ghiasvand R , Nissen‐Meyer LSH . Agaricus blazei‐Based Mushroom Extract Supplementation to Birch Allergic Blood Donors: A Randomized Clinical Trial. Nutrients. 2019;11(10):2339. 10.3390/nu11102339 PMC6836217 31581605

[14] Huang C , Wang Y , Li X , et al. Clinical features of patients infected with 2019 novel coronavirus in Wuhan, China. Lancet. 2020;395:497‐506. 31986264 10.1016/S0140-6736(20)30183-5 PMC7159299

[15] Nicholls JM , Poon LL , Lee KC , et al. Lung pathology of fatal severe acute respiratory syndrome. Lancet. 2003;361(9371):1773‐1778. 10.1016/s0140-6736(03)13413-7 12781536 PMC7112492

[16] Wong CK , Lam CW , Wu AK , et al. Plasma inflammatory cytokines and chemokines in severe acute respiratory syndrome. Clin Exp Immunol. 2004;136(1):95‐103. 10.1111/j.1365-2249.2004.02415.x 15030519 PMC1808997

[17] Openshaw PJM , Chiu C , Culley FJ , Johansson C . Protective and harmful immunity to RSV infection. Ann Rev Immunol. 2017;35:501‐532. 28226227 10.1146/annurev-immunol-051116-052206

[18] Wan Z , Zhou Z , Liu Y , et al. Regulatory T cells and T helper 17 cells in viral infection. Scand J Immunol. 2020;91:e12873. 10.1111/sji.12873 32090360

[19] Richardson JP , Moyes DL , Ho J , Naglik JR . Candida innate immunity at the mucosa. Semin Cell Dev Biol. 2019;89:58‐70. 10.1016/j.semcdb.2018.02.026 29501618

[20] Li G , Fan Y , Lai Y , et al. Coronavirus infections and immune responses. J Med Virol. 2020;92:424‐432. 10.1002/jmv.25685 31981224 PMC7166547

[21] Johnson E , Førland DT , Saetre L , Bernardshaw SV , Lyberg T , Hetland G . Effect of an extract based on the medicinal mushroom Agaricus blazei murill on release of cytokines, chemokines and leukocyte growth factors in human blood ex vivo and in vivo. Scand J Immunol. 2009;69(3):242‐250. 10.1111/j.1365-3083.2008.02218.x 19281536

[22] Ellertsen LK , Hetland G . An extract of the medicinal mushroom Agaricus blazei Murill can protect against allergy. Clin Mol Allergy. 2009;7:6. 10.1186/1476-7961-7-6 19416507 PMC2688003

[23] Førland DT , Johnson E , Saetre L , Lyberg T , Lygren I , Hetland G . Effect of an extract based on the medicinal mushroom Agaricus blazei Murill on expression of cytokines and calprotectin in patients with ulcerative colitis and Crohn's disease. Scand J Immunol. 2011;73(1):66‐75. 10.1111/j.1365-3083.2010.02477.x 21129005

[24] Johnson E , Førland DT , Hetland G , Sætre L , Olstad OK , Lyberg T . Effect of AndoSan™ on expression of adhesion molecules and production of reactive oxygen species in human monocytes and granulocytes in vivo. Scand J Gastroenterol. 2012;47(8–9):984‐992. 10.3109/00365521.2012.660544 22564240

[25] Sorimachi K , Ikehara Y , Maezato G , et al. Inhibition by Agaricus blazei Murill fractions of cytopathic effect induced by western equine encephalitis (WEE) virus on VERO cells in vitro. Biosci Biotechnol Biochem. 2001;65(7):1645‐1647. 10.1271/bbb.65.1645 11515550

[26] Faccin LC , Benati F , Rincão VP , et al. Linhares antiviral activity of aqueous and ethanol extracts and of an isolated polysaccharide from Agaricus Brasiliensis against poliovirus type 1. Lett Appl Microbiol. 2007;45(1):24‐28. 10.1111/j.1472-765X.2007.02153.x 17594456

[27] Hsu CH , Hwang KC , Chiang YH , Chou P . The mushroom Agaricus blazei Murill extract normalizes liver function in patients with chronic hepatitis B. J Altern Complement Med. 2008;14(3):299‐301. 10.1089/acm.2006.6344 18370584

[28] Grinde B , Hetland G , Johnson E . Effects on gene expression and viral load of a medicinal extract from Agaricus blazei in patients with chronic hepatitis C infection. Int Immunopharmacol. 2006;6:1311‐1314. 16782544 10.1016/j.intimp.2006.04.005

[29] Gu CQ , Li JW , Chao FH . Inhibition of Hepatitis B Virus by D‐fraction From Grifola Frondosa: Synergistic Effect of Combination With Interferon‐Alpha in HepG2 2.2.15. Antiviral Res. 2006;72(2):162‐165. 10.1016/j.antiviral.2006.05.01122 16846649

[30] Gu CQ , Li JW , Chao F , et al. Isolation, identification and function of a novel anti‐HSV‐1 protein from Grifola frondosa. Antiviral Res. 2007;75(3):250‐257. 10.1016/j.antiviral.2007.03.011 17475344

[31] Minari MC , Rincão VP , Soares SA , Ricardo NM , Nozawa C , Linhares RE . Antiviral properties of polysaccharides from Agaricus brasiliensis in the replication of bovine herpesvirus 1. Acta Virol. 2011;55(3):255‐259. 10.4149/av_2011_03_255 21978159

[32] Yamamoto KA , Galhardi LC , Rincão VP , et al. Antiherpetic activity of an Agaricus brasiliensis polysaccharide, its sulfated derivative and fractions. Int J Biol Macromol. 2013;52:9‐13. 10.1016/j.ijbiomac.2012.09.029 23043759

[33] Cardozo FT , Larsen IV , Carballo EV , et al. In vivo anti‐herpes simplex activity of a sulfated derivative of Agaricus brasiliensis mycelial polysaccharide. Antimicrob Agents Chemother. 2013;57(6):2541‐2549. 10.1128/AAC.02250-12 23507287 PMC3716167

[34] Zhao C , Gao L , Wang C , et al. Structural characterization and antiviral activity of a novel heteropolysaccharide isolated from Grifola frondosa against enterovirus 71. Carbohydr Polym. 2016;144:382‐389. 10.1016/j.carbpol.2015.12.005 27083830

[35] Avtonomova AV , Krasnopolskaya LM . Antiviral properties of basidiomycetes metabolites *Antibiot Khimioter* . Review [Article in Russian]. 2014; 59(7‐8):41‐48. 25975107

[36] Eguchi N , Fujino K , Thanasut K , et al. In vitro anti‐influenza virus activity of Agaricus brasiliensis KA21. Biocontrol Sci. 2017;22(3):171‐174. 10.4265/bio.22.171 28954960

[37] Wu Y , Jiang H , Zhu E , et al. Hericium erinaceus polysaccharide facilitates restoration of injured intestinal mucosal immunity in Muscovy duck reovirus‐infected Muscovy ducklings. Int J Biol Macromol. 2018;107:1151‐1161. 10.1016/j.ijbiomac.2017.09.092 28951299

[38] Ellan K , Thayan R , Raman J , Hidari KIPJ , Ismail N . Sabaratnam V . Anti‐viral activity of culinary and medicinal mushroom extracts against dengue virus serotype 2: an in‐vitro study. BMC Complement Altern Med. 2019;19(1):260. 10.1186/s12906-019-2629-y 31533688 PMC6751638

[39] Bernardshaw S , Johnson E , Hetland G . An extract of the mushroom Agaricus blazei Murill administered orally protects against systemic Streptococcus pneumoniae infection in mice. Scand J Immunol. 2005;62(4):393‐398. 10.1111/j.1365-3083.2005.01667.x 16253127

[40] Bernardshaw S , Hetland G , Grinde B , Johnson E . An extract of the mushroom Agaricus blazei Murill protects against lethal septicemia in a mouse model of fecal peritonitis. Shock. 2006;25(4):420‐425. 10.1097/01.shk.0000209526.58614.92 16670646

[41] Wang L , He W , Yu X , et al. Coronavirus disease 2019 in elderly patients: Characteristics and prognostic factors based on 4‐week follow‐up. J Infect. 2020;80(6):639‐645. 10.1016/j.jinf.2020.03.019 32240670 PMC7118526

[42] Haak BW , Wiersinga WJ . The role of gut microbiota in sepsis. Lancet Gastroenterol Hepatol. 2017;2(2):135‐143. 10.1016/S2468-1253(16)30119-4 28403983

[43] Premnath P , Reck M , Wittstein K , Stadler M , Wagner‐Döbler I . Screening for inhibitors of mutacin synthesis in Streptococcus mutans using fluorescent reporter strains. BMC Microbiol. 2018;18(1):24. 10.1186/s12866-018-1170-3 29580208 PMC5870221

[44] Soković M , Ćirić A , Glamočlija J , Nikolić M , van Griensven LJ . Agaricus blazei hot water extract shows anti quorum sensing activity in the nosocomial human pathogen Pseudomonas aeruginosa. Molecules. 2014;19(4):4189‐4199. 10.3390/molecules19044189 24705563 PMC6271851

[45] Valadares DG , Duarte MC , Ramírez L , et al. Prophylactic or therapeutic administration of Agaricus blazei Murill is effective in treatment of murine visceral leishmaniasis. Exp Parasitol. 2012;132(2):228‐236. 10.1016/j.exppara.2012.07.005 22824583

[46] de Jesus Pereira NC , Régis WC , Costa LE , et al. Evaluation of adjuvant activity of fractions derived from Agaricus blazei, when in association with the recombinant LiHyp1 protein, to protect against visceral leishmaniasis. Exp Parasitol. 2015;153:180‐190. 10.1016/j.exppara.2015.03.027. Epub 2015 Apr 3 25845753

[47] Val CH , Brant F , Miranda AS , et al. Effect of mushroom Agaricus blazei on immune response and development of experimental cerebral malaria. Malar J. 2015;11(14):311. 10.1186/s12936-015-0832-y PMC4531523 26260055

[48] He X , Du X , Zang X , et al. Extraction, identification and antimicrobial activity of a new furanone, grifolaone A, from Grifola frondosa. Nat Prod Res. 2016;30(8):941‐947. 10.1080/14786419.2015.1081197 26374926

[49] Liu JH , Li L , Shang XD , Zhang JL , Tan Q . Anti‐Helicobacter pylori activity of bioactive components isolated from Hericium erinaceus. J Ethnopharmacol. 2016;183:54‐58. 10.1016/j.jep.2015.09.004 26364939

[50] Wang G , Zhang X , Maier SE , Zhang L , In MRJ . Vitro and in vivo inhibition of *Helicobacter pylori* by ethanolic extracts of Lion's Mane Medicinal Mushroom, *Hericium erinaceus* (Agaricomycetes). Int J Med Mushrooms. 2019;21(1):1‐11. 10.1615/IntJMedMushrooms.2018029487 30806251

[51] Wang X‐Y , Yin J‐Y , Nie S‐P , Xie M‐Y . Isolation, purification and physicochemical properties of polysaccharide from fruiting body of Hericium erinaceus and its effect on colonic health of mice. Int J Biol Macromol. 2018; 107(Pt A):1310‐1319. 10.1016/j.ijbiomac.2017.09.112 28965966

[52] Croccia C , Agnaldo AJ , Ribeiro Pinto LF , et al. Royal Sun Medicinal Mushroom Agaricus Brasiliensis (Higher Basidiomycetes) and the Attenuation of Pulmonary Inflammation Induced by 4‐(methylnitrosamino)‐1‐(3‐pyridyl)‐1‐butanone (NNK). Int J Med Mushrooms. 2013;15(4):345‐355. 10.1615/intjmedmushr.v15.i4.20 23796216

[53] Lee KF , Chen JH , Teng CC , et al. Protective effects of Hericium erinaceus mycelium and its isolated erinacine A against ischemia‐injury‐induced neuronal cell death via the inhibition of iNOS/p38 MAPK and nitrotyrosine. Int J Mol Sci. 2014;15(9):15073‐15089. 10.3390/ijms150915073 25167134 PMC4200813

[54] Diling C , Xin Y , Chaoqun Z , et al. Extracts from Hericium erinaceus relieve inflammatory bowel disease by regulating immunity and gut microbiota. Oncotarget. 2017;8(49):85838‐85857. 10.18632/oncotarget.20689 29156761 PMC5689651

[55] Ren Y , Geng Y , Du Y , et al. Polysaccharide of Hericium erinaceus attenuates colitis in C57BL/6 mice via regulation of oxidative stress, inflammation‐related signaling pathways and modulating the composition of the gut microbiota. J Nutr Biochem. 2018;57:67‐76. 29677563 10.1016/j.jnutbio.2018.03.005

[56] Nakamura A , Zhu Q , Yokoyama Y , et al. Agaricus brasiliensis KA21 may prevent diet‐induced nash through its antioxidant, anti‐inflammatory, and anti‐fibrotic activities in the liver. Foods. 2019;8(11):546. 10.3390/foods8110546 PMC6915480 31689883

[57] Li L , Guo WL , Zhang W , et al. Grifola frondosa polysaccharides ameliorate lipid metabolic disorders and gut microbiota dysbiosis in high‐fat diet fed rats. Food Funct. 2019;10(5):2560‐2572. 10.1039/c9fo00075e 30994668

[58] Li IC , Lee LY , Chen YJ , et al. Erinacine A‐enriched Hericium erinaceus mycelia promotes longevity in Drosophila melanogaster and aged mice. PLoS One. 2019;14(5):e0217226. 10.1371/journal.pone.0217226 31100095 PMC6524823

[59] Ohno N , Furukawa M , Miura NN , et al. Antitumor beta glucan from the cultured fruit body of *Agaricus blazei* . Biol Pharm Bull. 2001;24:820‐828. 11456124 10.1248/bpb.24.820

[60] Takeyama T , Suzuki I , Ohno N , et al. Host‐mediated antitumor effect of grifolan NMF‐5N, a polysaccharide obtained from Grifola frondosa. J Pharmacobiodyn. 1987;10:644‐651. 3446772 10.1248/bpb1978.10.644

[61] Reynolds JA , Kastello MD , Harrington DG , et al. Glucan‐induced enhancement of host resistance to selected infectious diseases. Infect Immun. 1980;30:51‐57. 7439978 10.1128/iai.30.1.51-57.1980 PMC551275

[62] Hetland G , Ohno N , Aaberge IS , Lovik M . Protective effect of betaglucan against systemic streptococcus pneumoniae infection in mice. FEMS Immunol Med Microbiol. 2000;27:111‐116. 10640605 10.1111/j.1574-695X.2000.tb01420.x

[63] Chen L , Shao HJ , Su YB . Coimmunization of Agaricus blazei Murill extract with hepatitis B virus core protein through DNA vaccine enhances cellular and humoral immune responses. Int Immunopharmacol. 2004;4:403‐409. 15037217 10.1016/j.intimp.2003.12.015

[64] Chen L , Shao H . Extract from Agaricus blazei Murill can enhance immune responses elicited by DNA vaccine against foot‐and‐mouth disease. Vet Immunol Immunopathol. 2006;109:177‐182. 16213597 10.1016/j.vetimm.2005.08.028

[65] Takimoto H , Kato H , Kaneko M , Kumazawa Y . Amelioration of skewed Th1/Th2 balance in tumor‐bearing and asthma‐induced mice by oral administration of Agaricus blazei extracts. Immunopharmacol Immunotoxicol. 2008;30(4):747‐760. 10.1080/08923970802279092 18720167

[66] Levitz SM . Interactions of Toll‐like receptors with fungi. Microbes Infect. 2004;6:1351‐1355. 15596119 10.1016/j.micinf.2004.08.014

[67] Vetvicka V , Thornton BP , Ross GD . Soluble beta‐glucan polysaccharide binding to the lectin site of neutrophil or natural killer cell complement receptor type 3 (CD11b/CD18) generates a primed state of the receptor capable of mediating cytotoxicity of iC3b‐opsonized target cells. J Clin Invest. 1996;98:50‐61. 8690804 10.1172/JCI118777 PMC507400

[68] Takeuchi O , Akira S . Pattern recognition receptors and inflammation. Cell. 2010;140:805‐820. 20303872 10.1016/j.cell.2010.01.022

[69] Wu J , Zhang Y , Xin Z , Wu X . The crosstalk between TLR2 and NOD2 in Aspergillus fumigatus keratitis. Mol Immunol. 2015;64(2):235‐243. 10.1016/j.molimm.2014.11.021 25549945

[70] Thwe PM , Fritz DI , Snyder JP , et al. Syk‐dependent glycolytic reprogramming in dendritic cells regulates IL‐1β production to β‐glucan ligands in a TLR‐independent manner. J Leukoc Biol. 2019;106(6):1325‐1335. 10.1002/JLB.3A0819-207RR 31509298 PMC6883127

[71] Bensch KW , Raida M , Mägert HJ , Schulz‐Knappe P , Forssmann WG . hBD‐1: a novel beta‐defensin from human plasma. FEBS Lett. 1995;368(2):331‐335. 10.1016/0014-5793(95)00687-5 7628632

[72] Duits LA , Ravensbergen B , Rademaker M , Hiemstra PS , Nibbering PH . Expression of beta‐defensin 1 and 2 mRNA by human monocytes, macrophages and dendritic cells. Immunology. 2002;106(4):517‐525. 10.1046/j.1365-2567.2002.01430.x 12153515 PMC1782759

[73] Contreras G , Shirdel I , Braun MS , Wink M . Defensins: Transcriptional regulation and function beyond antimicrobial activity. Dev Comp Immunol. 2020;104:103556. 10.1016/j.dci.2019.103556 31747541

[74] Berven L , Karppinen P , Hetland G , Samuelsen ABC . The polar high molecular weight fraction of the *Agaricus blazei* Murill extract, AndoSan™, reduces the activity of the tumor‐associated protease, legumain, in RAW 264.7 cells. J Med Food. 2015;18(4):429‐438. 10.1089/jmf.2014.0018 25136950

[75] Hetland G , Tryggestad AMA , Espevik T , et al. The medicinal and antitumor mushroom Agaricus blazeii Murill activates NF‐kappaB via TLR2 in monocytic cells and induces expression of cell surface markers and production of cytokines in human monocyte‐ derived dendritic cells (MDDC) in vitro. Eur J Cancer Suppl. 2010;8(5):65. 10.1016/s1359-6349(10)71054-5

[76] Lu CC , Hsu YJ , Chang CJ , et al. Immunomodulatory properties of medicinal mushrooms: differential effects of water and ethanol extracts on NK cell‐mediated cytotoxicity. Innate Immun. 2016;22(7):522‐533. 10.1177/1753425916661402 27469258

[77] Liu Y , Zhang L , Zhu X , Wang Y , Liu W , Gong W . Polysaccharide Agaricus blazei Murill stimulates myeloid derived suppressor cell differentiation from M2 to M1 type, which mediates inhibition of tumour immune‐evasion via the Toll‐like receptor 2 pathway. Immunology. 2015;146(3):379‐391. 26194418 10.1111/imm.12508 PMC4610627

[78] Wells AI , Coyne CB . Enteroviruses: A gut‐wrenching game of entry, detection, and evasion. Viruses. 2019;11(5):460. 10.3390/v11050460 PMC6563291 31117206

[79] Wang SM , Lei HY , Liu CC . Cytokine immunopathogenesis of enterovirus 71 brain stem encephalitis. Clin Dev Immunol. 2012;2012:876241. 10.1155/2012/876241 22956971 PMC3432373

[80] Tognarelli EI , Palomino TF , Corrales N , Bueno SM , Kalergis AM , González PA . Herpes simplex virus evasion of early host antiviral responses. Front Cell Infect Microbiol. 2019;9:127. 10.3389/fcimb.2019.00127 31114761 PMC6503643

[81] Chen X , Zheng M , Huang M , et al. Muscovy duck reovirus infection disrupts the composition of intestinal microbiota in muscovy ducklings. Curr Microbiol. 2020;77(5):769‐778. 10.1007/s00284-019-01865-8 31919671

[82] Murphy PM . Viral exploitation and subversion of the immune system through chemokine mimicry. Nat Immunol. 2001;2(2):116‐122. 10.1038/84214 11175803

[83] McGeer PL , Lee M , McGeer EG . A review of human diseases caused or exacerbated by aberrant complement activation. Neurobiol Aging. 2017;52:12‐22. 10.1016/j.neurobiolaging.2016.12.017 28104543

[84] Morgan BP . Complement in the pathogenesis of Alzheimer’s disease. Semin Immopathol. 2018;40:113‐124. 10.1007/s00281-017-0662-9 PMC5794825 29134267

[85] Sina C , Kemper C , Derer S . The intestinal complement system in inflammatory bowel disease: Shaping intestinal barrier function. Semin Immunol. 2018;37:66‐73. 10.1016/j.smim.2018.02.008 29486961

[86] Tzeng TT , Chen CC , Chen CC , et al. The cyanthin diterpenoid and sesterterpene constituents of Hericium erinaceus mycelium ameliorates Alzheimer’s disease‐related pathologies in APP/PS1 transgenic mice. Int J Mol Sci. 2018;19(2):598. 10.3390/ijms19020598 PMC5855820 29463001

[87] Shimizu S , Kitada H , Yokota H , et al. Activation f the alternative complement pathway by Agaricus blazei Murill. Phytomedicine. 2002;9:536‐545. 10.1078/09447110260573047 12403163

[88] Arbore G , Kemper C , Kolev M . Intracellular complement − the complosome − in immune cell regulation. Mol Immunol. 2017;89:2‐9. 10.1016/j.molimm.2017.05.012 28601357 PMC7112704

[89] Hetland G , Eskeland T . Formation of the functional alternative pathway of complement by human monocytes in vitro as demonstrated by phagocytosis of agarose beads. Scand J Immunol. 1986;23(3):301‐308. 3952470 10.1111/j.1365-3083.1986.tb01971.x

[90] Johnson E , Hetland G . Mononuclear phagocytes have the potential to synthesize the complete functional complement system. Scand J Immunol. 1988;27(5):489‐493. 3287594 10.1111/j.1365-3083.1988.tb02375.x

[91] Hetland G , Eide DM , Tangen JM , Haugen MH , Mirlashari MR , Paulsen JE . The Agaricus blazei‐based mushroom extract, Andosan™, protects against intestinal tumorigenesis in the A/J Min/+ mouse. PLoS One. 2016;11(12):e0167754. 10.1371/journal.pone.0167754 28002446 PMC5176274

[92] Paul S , Singh AK , Shilpi LG . Phenotypic and functional plasticity of gamma‐delta (γδ) T cells in inflammation and tolerance. Int Rev Immunol. 2014;33(6):537‐558. 10.3109/08830185.2013.863306 24354324

[93] Egli A , Santer DM , O'Shea D , Tyrrell DL , Houghton M . The impact of the interferon‐lambda family on the innate and adaptive immune response to viral infections. Emerg Microbes Infect. 2014;3(7):e51. 10.1038/emi.2014.51 26038748 PMC4126180

[94] Zhou JH , Wang YN , Chang QY , Ma P , Hu Y , Cao X . Type III interferons in viral infection and antiviral immunity. Cell Physiol Biochem. 2018;51(1):173‐185. 10.1159/000495172 30439714

[95] Roberfroid M , Gibson GR , Hoyles L , et al. Prebiotic effects: metabolic and health benefits. Br J Nutr. 2010;104(Suppl 2):S1‐S63. 10.1017/S0007114510003363 20920376

[96] Lao EJ , Dimoso N , Raymond J , Mbega ER . The prebiotic potential of brewers' spent grain on livestock's health: a review. Trop Anim Health Prod. 2020;52(2):461‐472. 10.1007/s11250-019-02120-9 31898030

[97] Samuelsen AB , Schrezenmeir J , Knutsen SH . Effects of orally administered yeast‐derived beta‐glucans: a review. Mol Nutr Food Res. 2014;58(1):183‐193. 10.1002/mnfr.20130033 24019098

[98] Batbayar S , Lee DH , Kim HW . Immunomodulation of fungal β‐glucan in host defense signaling by dectin‐1. Biomol Ther. (Seoul). 2012;20(5):433‐445. 10.4062/biomolther.2012.20.5.433 24009832 PMC3762275

